# Molecular phylogeny of *Myxobolus orissae* (Myxosporea: Myxobolidae) infecting the gill lamellae of mrigal carp *Cirrhinus mrigala* (Actinopterygii: Cyprinidae)

**Published:** 2015-01

**Authors:** Thangapalam Jawahar Abraham, Sayani Banerjee, Avijit Patra, Agniswar Sarkar, Harresh Adikesavalu, Gadadhar Dash

**Affiliations:** Department of Aquatic Animal Health, Faculty of Fishery Sciences, West Bengal University of Animal and Fishery Sciences, Kolkata, West Bengal, India

**Keywords:** *Cirrhinus mrigala*, *Myxobolus orissae*, Molecular characterization, Phylogenetic relationship

## Abstract

Myxosporeans are best known for the diseases they cause in commercially important fish species. Identification of myxosporeans at the species-level is mainly based on conventional methods. The 18S rRNA gene sequence of morphologically identified *Myxobolus orissae *infecting the gill lamellae of mrigal carp *Cirrhinus mrigala *was characterized in the present study. The plasmodia of *M.* *orissae* were small, elongated and white to pale in colour. Phylogenetically, the 18S rDNA nucleotide sequence of *M. orissae* was clustered with other gill-infecting *Myxobolus* spp. of cyprinids. The species closely related to *M. orissae* was *M. koi *(FJ841887) infecting the gill lamellae of *Cyprinus carpio *with 96% similarity. The carp fin-infecting *Thelohanellus caudatus *(KC865607) from India exhibited only 78% DNA sequence similarity with *M. orissae. *Low level of *M. orissae* infection on gill caused thickening of epithelial cells surrounding the plasmodium. Under stressful conditions, it is likely that such infection can easily spread in confined fish and may cause serious disease outbreaks and economical losses.

## INTRODUCTION

The phylum Myxozoa has more than 2100 species in 58 genera to date and is divided into two classes, Myxosporea and Malacosporea [1]. Myxozoans are spore-forming parasites of both freshwater and marine fish and have been reviewed extensively [1-5]. Identification of myxozoans at the species-level is mainly based on the number of valves and polar capsules, arrangement of the polar capsules, ornamentation of spores and spore dimensions by conventional methods [5]. Smothers *et al*. [6] were the first to use ribosomal DNA (rDNA) sequence analysis to study the phylogeny of Myxozoa. Since then, small subunit rDNA sequences of myxozoans have been employed to address systematics and life cycle questions, and for the development of highly sensitive and specific diagnostic tests. The inclusion of sequence information is now a requirement in taxonomic and phylogenetic studies [3, 7-10]. 

 Myxosporeans are best known for the diseases they cause in commercially important fish [4, 5, 11]. Even though most of them are not harmful to fish, some species cause diseases. With the expansion in freshwater aquaculture, several myxosporeans have been found to be important pathogens [5]. Although the morphological data on many *Myxobolus *spp. from India are available [4], the molecular data are lacking. Recently, we reported the molecular characterization of fin-infecting* Thelohallenus caudatus* (accession number KC865607) from *Labeo rohita *(Hamilton) [12]. We also characterized *Myxobolus catmrigalae *(accession number KC933944) and *M. cuttacki* (accession number KF465682) infecting the gill lamellae of mrigal carp *Cirrhinus mrigala *(Hamilton) (Unpublished) and bata *Labeo bata *(Hamilton) [13], respectively*. *This communication reports the molecular phylogeny of conventionally identified *M. orissae *infecting the gill lamellae of *C. mrigala*.

## MATERIALS AND METHODS

**Microscopy and Morphometry: **A *Myxobolus* species infecting the gill lamellae of mrigal carp *Cirrhinus mrigala* collected from Garia (Lat. 22°27’59’’N; Long. 88°24’18’’E), South 24 Parganas district, West Bengal, India during the routine survey work on parasitic infection of cultured carps in March 2013 was characterized by morphometric and molecular techniques. The cultured sub-adult *C. mrigala* (n=60) of weight 150-210 g were brought to the laboratory within an hour of collection in oxygen filled polythene bags. In the laboratory, the gills on both sides of carp were removed and placed in separate Petri-dishes containing filtered water and examined thoroughly for myxosporean parasites. The parasitic frequency index (PFI) was calculated by taking the number of hosts infected by myxosporean parasite against the total number of hosts examined. Further, three cyst-like plasmodia of similar morphology found on single gill lamella of mrigal carp were collected for morphometric and molecular characterization. Guidelines of Lom and Arthur [14] were followed for the morphometric characterization of myxosporean. In brief, a fresh plasmodium was first taken on clean grease free glass slide with few drops of distilled water and slightly ruptured. The spores released from the plasmodium were then spread onto clean grease free glass slides, covered with cover slips and sealed with Distrene, Plasticizer and Xylene (DPX) for examination under oil immersion (100X) lens. Two fresh spore smears were treated with 2% KOH (w/v) for polar filament extrusion. The Indian ink method [15] was employed for observing the mucous membrane around the spores. Smears of fresh spores were treated with Lugol’s iodine solution to observe iodinophilic vacuoles in the sporoplasm. For permanent slides, air dried smears were fixed with acetone free absolute methanol for about 8 min and finally stained with Giemsa solution for 40 min. Giemsa solution was prepared by dissolving 0.5 g Giemsa powder in 33 mL glycerol at 50-60ºC for 90 min in a water bath followed by the addition of 33 mL methanol. This solution was matured in dark for 15 days and diluted with phosphate buffer (pH 7.2) in the ratio of 1:2 prior to use. The slides containing myxosporean spores were observed under oil immersion (100X) lens of Motic BA400 microscope with inbuilt digital camera. Morphometric measurements in μm were done by Motic Image Plus Version2 software. The gill of infected carp was fixed in Bouin's solution for 24 h. The fixed gill was prepared histologically using standard techniques, embedded in paraffin wax and 5 μm sections prepared and stained with haematoxylin and eosin.

**DNA extraction, PCR Amplification and Sequencing** **: **Molecular characterization of morphologically identified *Myxobolus* *orissae* was done as described in Mondal *et al*. [12]. The DNA was extracted from the second plasmodium collected from the same gill lamella of mrigal carp. After morphometric confirmation with the spores of first plasmodium, the spores were suspended in 500 µL lysis buffer (100 mM NaCl, 10 mM Tris, 10 mM EDTA, 0.2% SDS, 0.4 mg/mL Proteinase K) and incubated overnight at 55°C. Then, 500 µL of phenol: chloroform (1:1) was added to the digested spores, mixed gently and centrifuged at 5200g for 10 min. The upper phase was transferred to a new tube and mixed with 1/10^th^ volume of sodium acetate (3 M, pH 5.2) and 2 volumes of 96% ethanol (Amresco, USA). The DNA was precipitated at –20°C overnight and pelleted by centrifugation at 10000g for 30 min. The pellet was washed once with 70% ethanol, air-dried for several minutes and resuspended in 30 µL of molecular biology grade water. 

 The universal eukaryotic primers -ERIB1, 5´-ACC TGG TTG ATC CTG CCA G-3´ and ERIB10, 5´-CTT CCG CAG GTT CAC CTA CGG-3´ [16] were used for the amplification of 18S rDNA by Eppendorf Master cycler Pro S. The PCR was run using a mixture containing 50 ng of genomic DNA, 10 μM of each primer, 2X PCR TaqMixture. Amplification was done by initial denaturation at 95°C for 5 min, followed by 35 cycles of denaturation at 95°C for 30 sec, annealing of primers at 51°C for 30 sec and extension at 72°C for 60 sec. The final extension was at 72°C for 5 min. The PCR products were analysed on a 1.5% agarose gel containing 0.5 μg/mL ethidium bromide in 1X Tris-acetate-EDTA (TAE) buffer. Following purification of amplified PCR product by EXO-SAP treatment, the DNA was quantified and subjected to automated DNA sequencing on ABI 3730xl Genetic Analyzer. Sequencing was carried out using BigDye® Terminator v3.1 Cycle sequencing kit (Applied Biosystems, USA) following manufacturers’ instructions. Electrophoresis and data analysis were carried out on the ABI 3730xl Genetic Analyzer. 

**Phylogenetic Analysis:** Phylogenetic analysis was performed on a selection of 18S rDNA sequences that comprised the new sequence (KF448527) and 25 additional sequences from closely related species available in NCBI Genbank database using the basic local alignment search tool (BLAST) and other representatives of the Myxobolidae clade as described by Fiala [8]. *Ceratomyxa shasta* (AF001579) isolated from *Oncorhynchus mykiss* (Walbaum) was used as an out-group. Genetic distance analyses were conducted using the Kimura 2-parameter model [17] in MEGA5 [18]. Included codon positions were 1st+2^nd^+3^rd^+Noncoding. All positions containing gaps and missing data were eliminated. The Bayesian phylogenetic analysis was conducted using MrBayes v3.2.2 [19]. Sequence alignment was performed by **MU**ltiple **S**equence **C**omparison by **L**og**-** **E**xpectation (MUSCLE) and maximum likelihood (ML) phylogenetic tree was generated in TreeDyn mode (http://phylogeny.lirmm.fr). A total of 10,000 generation was taken for phylogenetic tree, which provided the Bayesian posterior probabilities and bootstrap values in each branch, and was proportional to the number of substitutions per site [19].

## RESULTS AND DISCUSSION

**Figure 1:**
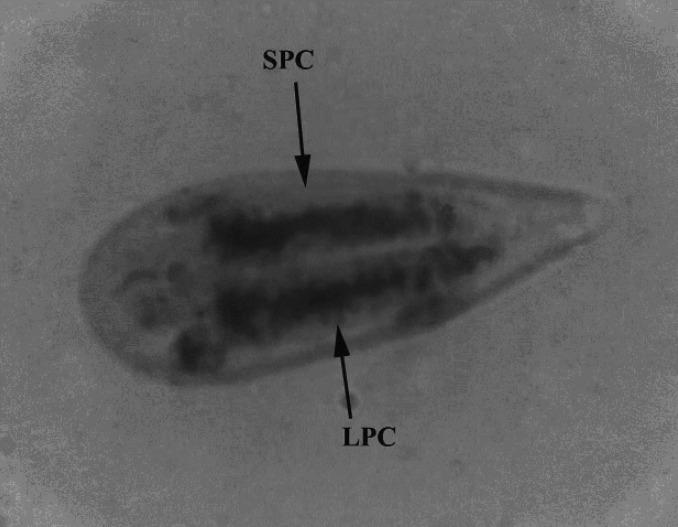
Mature spore of Giemsa stained gill-infecting *Myxobolus orissae* (1000X) from mrigal carp *Cirrhinus mrigala. *(LPC= Larger polar capsule, SPC= Smaller polar capsule).

**Morphometry of** ***Myxobolus orissae*:** Of the 60 mrigal carp screened, 39 (PFI = 65%) had gill myxosporeans with very low to moderate infection. The isolated cyst-like plasmodia from the single gill lamella of mrigal carp were small, elongated and white to pale in colour. The spores (n = 20) measured 15.6-19.7 (17.3±1.0) μm in length (L) and 5.7-9.3 (6.7±0.7) μm in breadth (B). They were elongated and pyriform in shape (Fig. 1). The anterior end was flat between the openings of polar capsules and posterior end was broad rounded. Shell valves were thick, symmetrical and smooth without any parietal folds. A distinct intercapsular appendix was present at the anterior end. Two polar capsules were distinctly unequal. The large polar capsules measured 6.8-13.5 (8.8±3.9) μm L x 1.4-3.1 (1.9±0.9) μm B. The smaller ones were 6.9-11.5 (7.4±3.2) μm L x 1.7-2.4 (1.6±0.7) μm B in size. Both were broadly pyriform with pointed anterior end and rounded posterior end. The capsules opened independently. Both polar capsules were situated parallel to each other in spore cavity. The polar filaments of large and small capsules were observed to reach 91-131 μm in length. They had very thick base and very thin edges.

**Figure 2:**
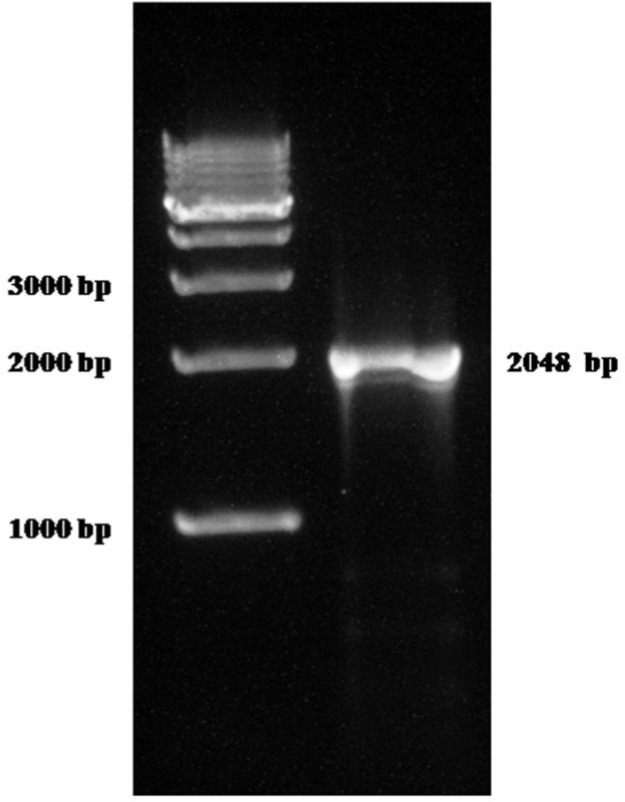
Agarose gel (1.5%) showing 18S rDNA gene amplification of *Myxobolus orissae* from mrigal carp *Cirrhinus mrigala*

**Figure 3:**
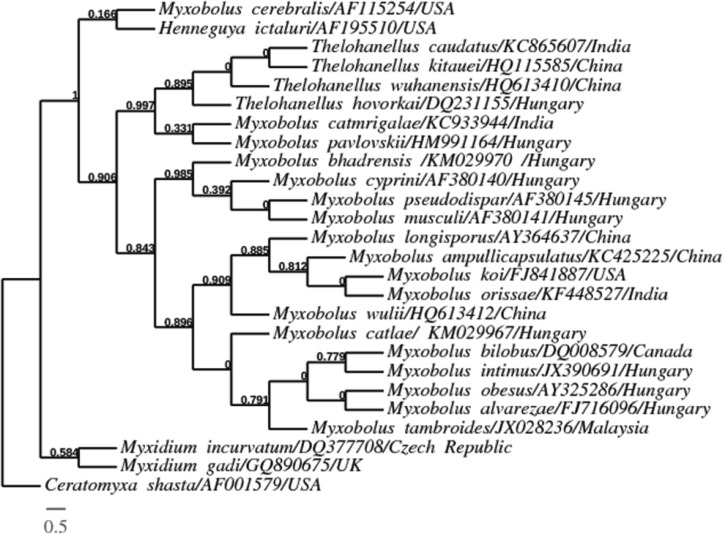
Molecular phylogenetic tree produced by Bayesian analysis. Numbers at the branches are Bayesian posterior probabilities. The numbers after taxa refer to accession numbers in GenBank. Geographical location is provided for each species. Scale bar = amount of inferred evolutionary change along the branch lengths

**Molecular comparison: **The universal eukaryotic primer sets ERIB1 and ERIB10 successfully amplified approximately 2048 bp fragments of the 18S rRNA gene from *M. orissae *(Fig. 2). The consensus nucleotide sequence with 1766 bp (edited sequence) was deposited in NCBI GenBank under the accession number KF448527. Phylogenetically, the 18S rDNA nucleotide sequence of gill infecting *M. orissae *was clustered with other *Myxobolus *spp. infecting cyprinid gills. All the skeletal muscle infecting *M. cyprini *(AF380140), *M. musculi *(AF380141) and *M. pseudodispar* (AF380145), and kidney infecting *M. bhadrensis *(KM029970) were distinctly different from the gill-infecting *Myxobolus *spp. and clustered together as a separate subclade. Other representatives of the Myxobolidae clade such as *Henneguya, Myxidium* and *Thelohanellus* were also distinctly different from the gill-infecting *Myxobolus *spp. and clustered separately. The out group *C. shasta* (AF001579) was phylogenetically clustered distinctly as a separate lineage (Fig. 3). The similarity among the 18S rDNA sequence of *M. orissae* and other representative myxosporeans was 78-96%. Evolutionary pair-wise distances among *M. orissae* and other analyzed myxosporean species measured by Kimura-2-parameter algorithm, ranged from 0.002 for *M. koi* (FJ841887) to 0.37 for (*T. caudatus* KC865607). The evolutionary pair-wise distance between the gill lamellae infecting *M.* *catmrigalae* and *M. orissae* was 0.24 (Table 2).

Over the years, the list myxosporean parasites and their morphometry reported from India have increased [4, 20, 21]. This study provided the molecular data on gill lamellae infecting *M. orissae *for the first time*,* whose identity on the basis of morphometry had been described [22]. Myxosporeans are characterized as host, organ and tissue specific organisms [23]. According to him, host and infection site are important characters for specific assignment. As shown in Table 1, length of the spore (LS) and breadth of the spore (BS) ratio (1: 0.39) as well as the large polar capsules length and breadth (LLPC and BLPC) ratio (1: 0.21) and small polar capsules length and breadth (LSPC and BSPC) ratio (1: 0.21) are in conformity with the original descriptions of *M. orissae*, LS: BS = 1: 0.43; LLPC: BLPC = 1: 0.20; and LSPC: BSPC = 1: 0.33 [22] and differed from several other gill-infecting *Myxobolus *spp. with unequal polar capsules from Indian cyprinids [4]. These observations, thus, confirmed that the *Myxobolus* species found on the gill lamella of mrigal carp was *M. orissae* in its morphology, host (carp) specificity and tissue (gill) tropism. 

**Table 1:** Spore morphometry and index of *Myxobolus orissae* infecting the gill lamellae of mrigal carp, *Cirrhinus mrigala*

**Characters**	**Range**	**Mean** **±SD**
Length of the spore, µm (LS)	15.60 - 19.70	17.25±0.98
Breadth of the spore, µm (BS)	5.70 - 9.30	6.70±0.78
Length of small polar capsule, µm (LSPC)	6.90 - 11.50	7.44±3.22
Breadth of small polar capsule, µm (BSPC)	1.70 - 2.40	1.57±0.74
Length of large polar capsule, µm (LLPC)	6.80 - 13.50	8.75±3.85
Breadth of large polar capsule, µm (BLPC)	1.40 - 3.10	1.90±0.89
Length of nucleus, µm	2.50	2.50±0.00
Spore index	Index	
LS:BS	1:0.39	--
LLPC:BLPC	1:0.21	--
LSPC:BSPC	1:0.21	--
LLPC:LSPC	1:0.85	--
BLPC:BSPC	1:0.82	--

**Table 2:** Similarity of 18S rRNA gene sequences of selected myxozoan species available in NCBI GenBank and *Myxobolus orissae* and estimates of evolutionary divergence between the sequences of Myxosporea available in NCBI GenBank database

	**Myxosporean species**	**number**	**DNA similarity**	**1**	**2**	**3**	**4**	**5**	**6**	**7**	**8**	**9**	**10**	**11**	**12**	**13**	**14**	**15**	**16**	**17**	**18**	**19**	**20**	**21**	**22**	**23**	**24**	**25**	**26**
1	*Myxobolus koi*	FJ841887	96	0.00																									
2	*Myxobolus orissae*	KF448527	-	0.002	**0.00**																								
3	*Myxobolus longisporus*	AY364637	93	0.03	**0.04**	0.00																							
4	*Myxobolus wulii*	HQ613412	92	0.03	**0.03**	0.03	0.00																						
5	*Myxobolus ampullicapsulatus*	KC425225	91	0.05	**0.05**	0.04	0.05	0.00																					
6	*Myxobolus catmrigalae*	KC933944	91	0.23	**0.24**	0.24	0.24	0.24	0.00																				
7	*Myxobolus pavlovskii*	HM991164	88	0.15	**0.15**	0.16	0.15	0.16	0.10	0.00																			
8	*Myxobolus intimus*	JX390691	92	0.07	**0.07**	0.08	0.08	0.08	0.21	0.16	0.00																		
9	*Myxobolus alvarezae*	FJ716096	92	0.07	**0.07**	0.07	0.07	0.08	0.21	0.16	0.02	0.00																	
10	*Myxobolus obesus*	AY325286	90	0.08	**0.08**	0.08	0.09	0.09	0.21	0.17	0.02	0.03	0.00																
11	*Myxobolus bilobus*	DQ008579	90	0.06	**0.06**	0.07	0.08	0.08	0.21	0.16	0.04	0.03	0.04	0.00															
12	*Myxobolus tambroides*	JX028236	89	0.08	**0.08**	0.08	0.08	0.08	0.22	0.16	0.04	0.04	0.05	0.05	0.00														
13	*Myxobolus cerebralis*	AF115254	85	0.17	**0.17**	0.15	0.18	0.17	0.24	0.19	0.18	0.17	0.18	0.19	0.19	0.00													
14	*Myxobolus musculi*	AF380141	87	0.13	**0.13**	0.14	0.14	0.14	0.22	0.17	0.10	0.11	0.12	0.11	0.11	0.18	0.00												
15	*Myxobolus pseudodispar*	AF380145	86	0.13	**0.14**	0.14	0.14	0.14	0.22	0.17	0.11	0.12	0.12	0.12	0.11	0.19	0.01	0.00											
16	*Myxobolus cyprini*	AF380140	86	0.13	**0.14**	0.14	0.14	0.15	0.21	0.16	0.10	0.11	0.12	0.11	0.11	0.18	0.01	0.02	0.00										
17	*Thelohanellus kitauei*	HQ115585	87	0.18	**0.18**	0.18	0.17	0.18	0.15	0.10	0.17	0.17	0.18	0.18	0.17	0.19	0.17	0.17	0.17	0.00									
18	*Thelohanellushovorkai*	DQ231155	87	0.17	**0.18**	0.17	0.17	0.17	0.14	0.11	0.16	0.16	0.17	0.17	0.16	0.19	0.17	0.17	0.16	0.02	0.00								
19	*Thelohanellus wuhanensis*	HQ613410	87	0.18	**0.18**	0.17	0.18	0.17	0.13	0.10	0.16	0.16	0.17	0.16	0.18	0.19	0.17	0.16	0.16	0.03	0.02	0.00							
20	*Thelohanellus caudatus*	KC865607	78	0.37	**0.37**	0.36	0.36	0.34	0.33	0.28	0.34	0.34	0.35	0.35	0.33	0.38	0.37	0.37	0.37	0.22	0.21	0.22	0.00						
21	*Henneguya ictaluri*	AF195510	86	0.25	**0.25**	0.25	0.24	0.24	0.29	0.25	0.24	0.24	0.25	0.25	0.24	0.17	0.24	0.25	0.25	0.24	0.25	0.25	0.40	0.00					
22	*Myxidium incurvatum*	DQ377708	85	0.31	**0.32**	0.29	0.31	0.31	0.36	0.31	0.30	0.29	0.29	0.30	0.31	0.26	0.32	0.33	0.32	0.33	0.32	0.33	0.50	0.30	0.00				
23	*Myxidium gadi*	GQ890675	83	0.31	**0.31**	0.28	0.30	0.30	0.32	0.28	0.28	0.28	0.28	0.29	0.28	0.26	0.30	0.30	0.30	0.29	0.28	0.29	0.47	0.24	0.09	0.00			
24	*Myxobolus catlae*	KM029967	92	0.07	**0.08**	0.09	0.08	0.10	0.22	0.16	0.07	0.06	0.08	0.07	0.07	0.18	0.11	0.11	0.11	0.16	0.15	0.16	0.34	0.23	0.32	0.12	0.00		
25	*Myxobolus bhadrensis*	KM029970	82	0.12	**0.12**	0.14	0.13	0.13	0.21	0.17	0.10	0.10	0.12	0.11	0.11	0.18	0.03	0.03	0.03	0.17	0.17	0.17	0.38	0.25	0.31	0.31	0.12	0.00	
26	*Ceratomyxa shasta*	AF001579	83	0.33	**0.33**	0.32	0.32	0.33	0.39	0.34	0.32	0.33	0.31	0.33	0.32	0.33	0.34	0.34	0.35	0.33	0.33	0.33	0.53	0.30	0.22	0.18	0.33	0.35	0.00

Comparison of consensus sequence of gill-infecting *M. orissae* with other gill-infecting *Myxobolus* spp. sequences available in GenBank database showed that they are indeed myxobolids. Phylogenetic cluster was established on the basis of consensus sequence of length 1766 bp. It was very similar to and defined topologies resembling those generated by Fiala [8]. The phylogenetic tree placed *M. orissae* within the gill-infecting *Myxobolus* spp. of freshwater clade. Among the 18S rRNA gene sequences of selected gill-infecting *Myxobolus* spp. from GenBank database, the species phylogenetically closely related to *M. orissae* was *M. koi *(FJ841887) infecting the gill lamellae of koi, *Cyprinus carpio *from USA with 96% similarity. The sequence similarity among the 18S rDNA of *M. orissae* and most of the gill-infecting *Myxobolus* spp was 88-96%. On the other hand, our previously characterized carp fin-infecting *T. caudatus *(KC865607) exhibited only 78% DNA sequence similarity with *M. orissae*. The observed evolutionary pair-wise distances among *M. orissae* and other analyzed species ranging from 0.002 to 0.37 indicated high genetic diversity among myxosporeans.

The pathology of most gill-infecting myxosporeans is largely unclear. Although 65% of the sampled mrigal carp harboured myxosporeans on gills with very low to moderate infection, neither mortalities nor major disease outbreaks were recorded. However, all the cohabiting major and minor carps in the pond exhibited growth retardation with poor body muscle ratio. Histologically, only thickening of gill epithelial cells surrounding the plasmodium was observed (data not shown). These observations inferred that the gill-infecting *M. orissae* and other myxosporeans had low rate of infectivity. It is likely that the infection can easily spread in fish when confined under stressful conditions and may cause serious disease outbreaks and significant economical losses. The earlier observations on increased mortalities due to *Myxobolus* spp. in cultured fish over the years [2, 5, 11] implied that the gill-infecting myxosporeans are an emerging threat to commercial freshwater aquaculture. 

Myxosporean parasites are the major component of aquatic biodiversity, and their monitoring is considered an essential element of the management of fish health. Measures such as complete draining of water, proper drying of pond bottom after every culture operation, maintaining optimal pond water and pond sediment quality, reducing the levels of definitive host (annelids) of myxosporean parasites, etc are warranted, which would help control gill-infecting myxosporeans *in *aquaculture. Further studies on pathogenicity of *M. orissae* and other *Myxobolus *spp. under different culture conditions are necessary to manage these parasites in freshwater aquaculture systems.

## Notes

**Acknowledgements**: The work was supported by the Indian Council of Agricultural Research, Government of India, New Delhi under the Niche Area of Excellence programme. The authors thank the Vice-Chancellor of the West Bengal University of Animal and Fishery Sciences, Kolkata for providing necessary infrastructure facility to carry out the work.
